# Supplementary figures and images for: LncRNA NR120519 Blocks KRT17 to Promote Cell Proliferation and Migration in Hypopharyngeal Squamous Carcinoma
Source: Cancers (Basel). 2023 Jan 18;15(3):603. doi: 10.3390/cancers15030603 (PMC9913485; doi:10.3390/cancers15030603)

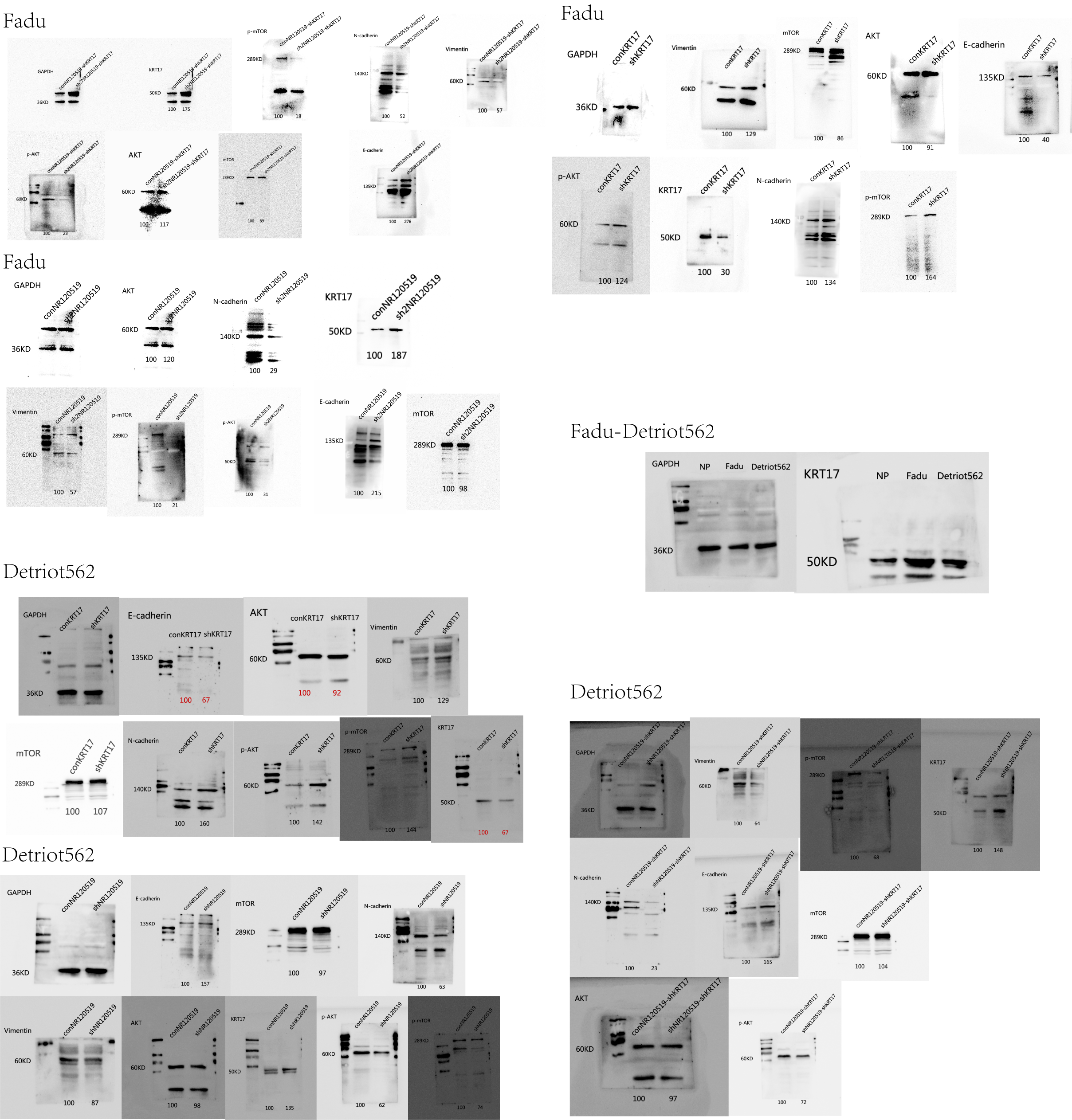

Supplement: Supplementary file 1 [file cancers-15-00603-s001.zip › wb-.tif]

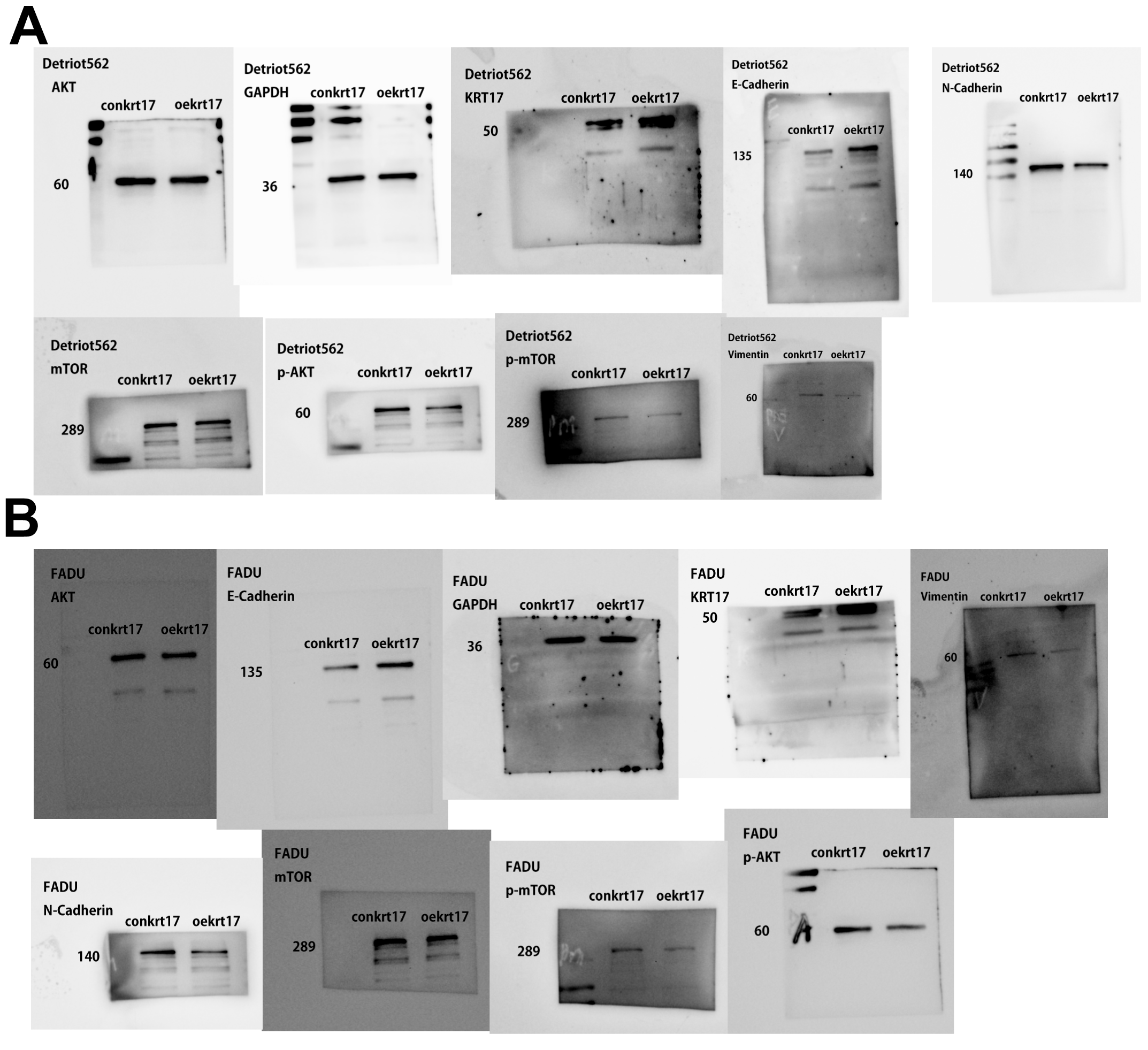

Supplement: Supplementary file 1 [file cancers-15-00603-s001.zip › WB.tif]
